# Influence of preoperative emotional state on postoperative pain following orthopedic and trauma surgery

**DOI:** 10.1590/0104-1169.0118.2481

**Published:** 2014

**Authors:** Gemma Robleda, Amalia Sillero-Sillero, Teresa Puig, Ignasi Gich, Josep-E Baños

**Affiliations:** 1 MSc, Researcher, Departamento de Metodología, Gestión Clínica e Investigación, Hospital de la Santa Creu i Sant Pau, Barcelona, Spain; 2 RN, Área Quirúrgica, Hospital de la Santa Creu i Sant Pau, Barcelona, Spain; 3 PhD, Researcher, Servicio de Epidemiologia Clínica, Institut d'Investigació Biomèdica Sant Pau, Hospital de la Santa Creu i Sant Pau, Universidad Autónoma de Barcelona, Spain; 4 PhD, Professor, Departamento de Ciencias Experimentales y de la Salud, Universitat Pompeu Fabra, Barcelona, Spain

**Keywords:** Pain, Postoperative, Anxiety, Surgery, Orthopedics

## Abstract

**OBJECTIVES:**

to analyze the relationship between preoperative emotional state and the prevalence and intensity of postoperative pain and to explore predictors of postoperative pain.

**METHOD:**

observational retrospective study undertaken among 127 adult patients of orthopedic and trauma surgery. Postoperative pain was assessed with the verbal numeric scale and with five variables of emotional state: anxiety, sweating, stress, fear, and crying. The Chi-squared test, Student's t test or ANOVA and a multivariate logistic regression analysis were used for the statistical analysis.

**RESULTS:**

the prevalence of immediate postoperative pain was 28%. Anxiety was the most common emotional factor (72%) and a predictive risk factor for moderate to severe postoperative pain (OR: 4.60, 95% CI 1.38 to 15.3, p<0.05, AUC: 0.72, 95% CI: 0.62 to 0.83). Age exerted a protective effect (OR 0.96, 95% CI: 0.94-0.99, p<0.01).

**CONCLUSION:**

preoperative anxiety and age are predictors of postoperative pain in patients undergoing orthopedic and trauma surgery.

## Introduction

Orthopedic and trauma surgeries are often associated with severe postoperative pain for two main reasons. First, the surgery often causes intense nociceptive stimulation of the musculoskeletal tissue^(^ 1 ^-^ 2 ^)^. Second, postoperative analgesia remains inadequate and exposes patients to severe postoperative pain^(^ 3 ^)^. This situation can lead to significant patient discomfort and negative physical and psychological effects as well as a major socio-economic impact by increasing the postoperative morbidity and hospital stay^(^ 4 ^)^. Timely prevention, detection and treatment may help reduce health problems associated with the high prevalence and intensity of postoperative pain.

Although postoperative pain is predictable and there are various ways to prevent and treat it, the correct approach widely varies from one patient to another in similar surgical procedures^(^ 5 ^)^. In this regard, knowing the emotional factors that influence the occurrence of postoperative pain would be an important contribution to a more appropriate treatment for each patient. It has been shown that certain emotional states such as anxiety and fear can influence individual responses to surgical intervention and to the need for analgesia^(^ 6 ^-^ 7 ^)^. In the last decade, there has been increasing interest in analyzing the influence of preoperative anxiety on postoperative pain and recovery, but the data come from heterogeneous studies that vary in their sociodemographic characteristics and the type of surgery^(^ 7 ^-^ 8 ^)^. Furthermore, studies on patients undergoing orthopedic surgery, one of the most painful procedures, are even scarcer^(^ 9 ^)^. Confirming the relationship between preoperative anxiety and postoperative pain is significant because if one exists, patients with high anxiety should be identified before surgery and should expect individualized strategies for pain treatment after surgery^(^ 7 ^,^ 10 ^)^. Patient expectations regarding surgery have changed, and the lack of postoperative pain is an important indicator of satisfaction with hospital care^(^ 11 ^,^ 12 ^)^.

In Spain, there are no recent data showing the prevalence of immediate postoperative pain following orthopedic or trauma surgery or the relationship between emotional factors and postoperative pain in these surgeries. This study was undertaken to analyze the relationship between preoperative emotional state and the prevalence and intensity of postoperative pain in patients undergoing orthopedic or trauma surgery as well as to explore potential predictors of postoperative pain.

## Methods

This analytical and retrospective observational study was conducted in the postoperative care unit (PCU) of a university hospital in Barcelona (Spain) between January and March 2011. The study protocol was approved by the Clinical Research Ethics Committee of the center (Code 36/2010), and the guidelines of the STROBE Statement were followed for observational studies^(^ 13 ^)^.

The study analyzed a convenience sample, selecting all patients who underwent trauma surgery (femoral fractures) or prosthetic surgery (hip, knee or shoulder arthroplasty) and met the inclusion and exclusion criteria. We included patients older than 18 years with a minimum stay of 4 hours in the PCU. This interval was chosen because at least four pain assessments were guaranteed during the immediate postoperative period, as pain is determined at least every hour during this period in routine clinical practice. Patients who could not perform the pain self-assessment (due to mechanical ventilation or cognitive impairment, for example) were excluded. These patients were easily identifiable because for the pain assessment of patients who were not able to describe pain, we used behavioral scales; the results from this assessment are included in the medical records. During the study period, 568 orthopedic and trauma surgery patients were included. In total, 171 were eligible, and 44 were excluded due to a PCU stay of less than 4 hours (16), cognitive impairment (14), mechanical ventilation (5) and incomplete data in the medical record (9). The final study sample consisted of 127 patients. This number allowed achieving the study objectives, assuming that the correlation coefficients of 0.2 with the calculation of Spearman's rank correlation coefficient would be significant and that this number could generate a multivariate analysis. A reasonable percentage of losses valued at 15% was recorded. A notebook was designed for specific data collection.

The primary endpoint was immediate postoperative pain (within the first hour of PCU admission), and the following secondary endpoints were assessed: emotional state (anxiety, stress, sweating, fear, crying); maximum postoperative pain in the PCU, maximum pain within the first 24 hours, average pain during the PCU stay, pain within the first 12 hours and 24 hours; demographic variables: age (>65 years, <65 years) and gender; previous physical condition: low-risk patients (American Society of Anesthesiologists [ASA]: I or II) and high-risk surgical patients (ASA > II); presence of comorbidity (diabetes mellitus, chronic obstructive pulmonary disease, heart disease, previous surgery, alcohol and/or opioid abuse, previous diseases evolving with pain, pain medication, anxiety or depression); operating time (<90 min, ≥90 min); surgical indication (thighbone fracture or knee, hip or shoulder prosthesis); type of anesthesia (regional, general, or combined); and pain treatment (analgesic consumption and rescue analgesia).

Pain intensity was assessed using the Numeric Rating Scale (NRS)^(^ 14 ^)^. This validated scale ranges from 0 (no pain) to 10 (maximum pain). For this study, two standardized pain intensities were established: mild pain (NRS≤3) and moderate-to-severe pain (NRS≥4). In cases where there was more than one record of immediate pain, its average value was calculated. In routine clinical practice, the surgical nurse who meets the patient in the preanesthesia area evaluates the preoperative emotional state.

Preoperative emotional state was assessed with the routine clinical practice tool. It is a dichotomous scale (absent=0 / present=1) of 5 items: anxiety, sweating, stress, fear and crying. With these items, the total score for emotional state, which may range from 0 (total absence of alteration of preoperative emotional state) to 5 (highest disturbance of preoperative emotional state), was obtained. For the purpose of this study, no levels of intensity of preoperative emotional state alterations were established.

Regarding the categorical variables, the results were presented as the number of cases and percentages. Concerning the quantitative variables, they were expressed as means ± standard deviations. The comparison of emotional state variables with immediate postoperative pain was performed with the Chi-squared test. Student's t test or ANOVA was used for quantitative variables, and calculation of Spearman's rank correlation coefficient was used with ordinal variables. With clinically relevant results that showed statistically significant differences in the bivariate analysis, a multivariate logistic regression analysis was performed. To validate this model, the Hoshmer-Lemeshow goodness-of-fit test was used. Additionally, as a discrimination index, the area under the curve (AUC) of the receiver operating characteristic (ROC) was used. The statistical analysis was performed using SPSS (version 19.0). The probability of making a Type I error at 5% (α=0.05) with a bilateral approach was set.

## Results

The general characteristics are summarized in Table 1 The mean age was 71±18 years (women: 77±13, men: 60±20, p<0.01), and 62% were women. In total, 60% underwent trauma surgery. The patients had a mean of 2.3±1.4 associated comorbidities, and 58% had low surgical risk (ASA I or II). During the PCU stay, 104 (82%) patients were given systemic analgesics, 15 (12%) had patient-controlled analgesia (PCA) by the epidural route, and 24 (19%) underwent regional anesthetic blocks. The most commonly used systemic analgesics were acetaminophen (76%), dexketoprofen (73%) and metamizol (62%). Thirty-one patients (24%) initiated regional analgesia in the PCU, mostly with ropivacaine and/or fentanyl. Rescue analgesia with intravenous bolus of morphine was required for moderate-to-severe pain in 31 patients (24%). The pain prevalence is presented in Table 2 and ranged from 28% during admission (95% CI; 21-35%) to 61% during the PCU stay (95% CI; 53-68%). Pain intensity was statistically higher in younger patients than in older patients (pain during admission: 1.9±2.3 versus 0.7±1.6, p<0.05, average pain in the PCU: 1.6±1.6 versus 1±1.2, p<0.05, maximum pain in the PCU: 4.2±3 versus 3±2.9, p<0.05, respectively).

**Table 1:** - General characteristics of the population (n=127), Barcelona, Spain, 2011

Variable			n	%	Mean	Standard Deviation
Gender						
	Women		79	62		
	Men		48	38		
Age, years					71	18
	Women				77	13
	Men				60,5	20
American Society of Anesthesiologists					2.3	0.7
	I		14	11		
	II		60	47		
	III		50	40		
	IV		2	2		
Operating time, min					128	53.5
	≤90 min		89	70		
	>90 min		38	30		
Type of anesthesia						
	General		20	15		
	Combined		21	17		
	Regional		86	68		
Type of surgery						
	Trauma surgery (fractures)		76	60		
	Prosthetic surgery		51	40		
	**	Knee replacement surgery	26	20		
	**	Hip replacement surgery	20	16		
	**	Shoulder replacement surgery	5	4		
Medical record						
	Diabetes mellitus		27	21		
	Heart disease		64	50		
	Chronic obstructive pulmonary disease		14	11		
	Previous surgery		84	66		
	Previous disease with chronic pain		35	44		
	Usual pain medication		30	38		
	Anxiety / depression		9	12		

**Table 2:** - Distribution of the pain prevalence according to its intensity (n=127), Barcelona, Spain, 2011

Intensity	% (n) with pain				
	Mean (standard deviation)	Total pain*	95% CI	Mild	Moderate to severe
		n (%)	n (%)	n (%)	n (%)
Admission to the PCU	1 (1.2)	28 (36)	21 – 35	15 (20)	13 (16)
Average stay in the PCU	1.2 (1.3)	61 (77)	53 - 68	54 (67)	6.5 (10)
Pain at 12 hours	2.2 (2.4)	56 (71)	46.5 – 64.5	35 (45)	21 (27)
Pain at 24 hours	1.7 (2.3)	46 (59)	37 – 53.5	28 (36)	18 (23)

* Total pain = NRS>1; Mild = NRS of 1-3; Moderate-to-severe = NRS>4

Regarding the analysis of maximum pain during the PCU stay, the results showed that 58 patients (45%) had moderate-to-severe maximum pain with an average intensity of 6±1.7 (95% CI, range 5.6-6.4) and that within the first 24 hours, 84 patients (66%) had moderate-to-severe maximum pain with an average intensity of 5.8±1.7 (95% CI, range 5.4-6). During prosthetic surgery, the maximum pain intensity during the PCU stay and within the first 24 hours was statistically higher than that during the trauma surgery (PCU: 3.9±2.9 versus 2.9±2.9, p<0.05, 24 hours: 5±2 versus 4±2.6, p<0.05, respectively). Patients who underwent general anesthesia experienced more pain during their PCU stay than did patients who underwent combined or regional anesthesia (PCU admission: 2.3±2.5 versus 1.2±2 versus 0.7±1.5, p<0.01, average PCU stay: 2±1.6 versus 1.4±1.4 versus 0.9±1.2, p<0.01, respectively*).* No statistically significant difference was observed in pain intensity by gender, number of comorbidities or duration of surgery.

**Figure 1:**
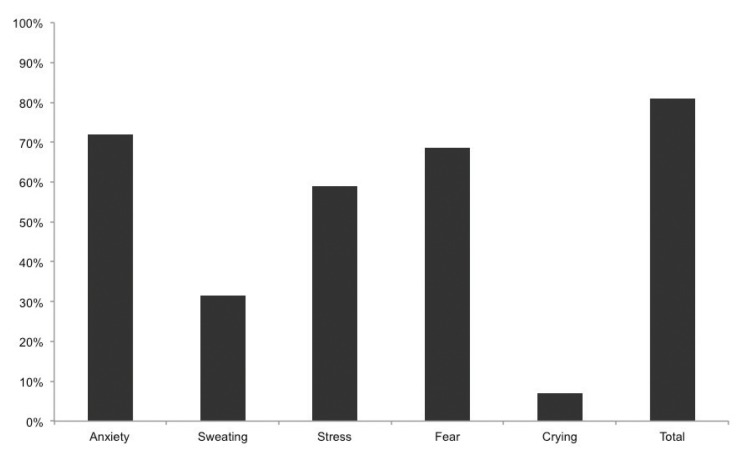
- Emotional state during the preoperative period (n=127)

In Figure 1, the results regarding the prevalence of preoperative emotional factors are described. Anxiety (72%), fear (68.5%) and stress (59%) were the most frequently detected indicators of altered emotional state. No significant differences were observed in the prevalence of emotional indicators by age group. Compared with men, women exhibited more stress (67% versus 46%, p<0.05) and crying (11% versus 0%, p<0.05). A trend without statistical significance was observed regarding the prevalence of anxiety and fear (anxiety: women 79% versus men 63%, p=0.06; fear: women 75% versus men 58%, p=0.08). As for surgery, patients undergoing prosthetic surgery displayed more preoperative anxiety than did patients undergoing trauma surgery (86% versus 63%, p<0.01).

The data for the association of immediate pain with emotional state are listed in Table 3. A statistically higher percentage of patients with preoperative anxiety showed immediate postoperative pain (anxiety: 89% with pain versus 66% with no pain, p<0.01). The results with respect to pain during the average PCU stay within the first 12 hours and 24 hours after surgery, as well as emotional factors, showed no significant differences.

**Table 3:** - Frequency of preoperative emotional factors depending upon immediate postoperative pain (n=127), Barcelona, Spain, 2011

	Pain (n=36)	No pain (n=91)	P
	n (%)	n (%)	
Anxiety	32 (89)	60 (66)	<0.01
Sweating	16 (44)	24 (26)	0.06
Stress	23 (64)	52 (57)	0.55
Fear	26 (72)	61 (67)	0.67
Crying	3 (8)	6 (7)	0.71
Total	33 (92)	70 (77)	0.28

Table 4 presents the results of the bivariate and multivariate analysis of factors associated with postoperative pain, the odds ratio values ​​and the statistical significance according to the logistic regression model. The multivariate analysis was performed using all variables that had significant clinical interest and with the data for immediate postoperative pain. The results revealed that age exerted a protective effect on postoperative pain (OR: 0.96, 95% CI: 0.94-0.99, p<0.001), whereas preoperative anxiety was a risk factor (OR: 4.60, 95% CI: 1.38-15.3, p<0.05). In the analysis of the ROC AUC, preoperative anxiety was established as a good predictive parameter for postoperative pain (AUC: 0.725, 95% CI: 0.62 to 0.83).

**Table 4:** - Bivariate and multivariate analysis according to the multiple logistic regression model of factors associated with immediate postoperative pain, Barcelona, Spain, 2011

		OR*	95% CI	p
Bivariate analysis				
	Age	0.962	0.940 – 0.985	<0.001
	General anesthesia	4.617	1.660 – 12.840	<0.05
	Combined anesthesia	1.619	0.545 – 4.808	<0.01
	Comorbidity: heart disease	3.043	1.332 – 6.953	<0.01
	Preoperative anxiety	4.592	1.382 – 15.262	<0.05
	Preoperative sweating	2.351	1.044 – 5.296	<0.05
Multivariate analysis				
	Age	0.962	0.940 – 0.985	<0.001
	Preoperative anxiety	4.592	1.382 – 12.34	<0.05

* Odds ratio

## Discussion

The main outcomes of this study reveal that nearly one out of three patients undergoing trauma or prosthetic surgery have pain during the immediate postoperative period and that more than half of these patients have moderate-to-severe pain within the first 24 hours of the postoperative period. The multivariate regression analysis also shows that there is an association between preoperative anxiety and postoperative pain.

In the last fifteen years, various clinical guidelines have been published with recommendations for treating postoperative pain^(^ 11 ^,^ 15 ^-^ 16 ^)^. Nevertheless, several authors agree on the need to improve pain therapy^(^ 3 ^,^ 17 ^)^. These shortcomings have been mentioned in this study, which reveals a prevalence of postoperative pain in trauma and prosthetic surgery ranging between 28% and 61%, with episodes of moderate-to-severe pain in 45% of patients during their PCU stay and 66% within the first 24 hours of the postoperative period. These findings are consistent with those of other studies where between 22% and 67% of patients of trauma surgery had pain within the first 24 hours of the postoperative period^(^ 3 ^,^ 18 ^)^ and 64% on the previous day. However, the authors did not report whether it was immediate postoperative pain^(^ 3 ^)^. Although the studies have reported a high prevalence of postoperative pain, comparing their results is difficult, hence the need to determine the influence of socio-demographic, clinical and psychological factors as predictors of postoperative pain^(^ 7 ^,^ 9 ^,^ 19 ^-^ 23 ^)^.

Patient characteristics such as age, gender, type of patient, previous experiences or type of surgery may help explain the large variability in the prevalence of postoperative pain. However, the literature is rather contradictory in these respects. In a systematic review of 23,000 patients, the authors highlighted preoperative pain, anxiety, age and type of surgery as the most significant predictors of postoperative pain and analgesic consumption^(^ 7 ^)^, but they disregarded gender as a consistent predictor. In another study, with a cohort of 1,990 patients, anxiety and depression were predictors of pain, but neither age nor gender were associated with pain^(^ 19 ^)^. Some authors have described a higher prevalence of pain among young people^(^ 23 ^)^, while others report a higher prevalence in older patients^(^ 20 ^,^ 22 ^)^. There are also mismatches in the prevalence of pain among men and women. Some authors observed more pain in women and younger patients while others found a higher prevalence in men^(^ 20 ^,^ 23 ^)^. Our results are consistent with some studies and differ from others. Young patients experienced more pain during the immediate postoperative period, but no differences according to gender or other factors such as operating time or comorbidities were observed. Another factor associated with a greater prevalence of pain is the type of surgery^(^ 18 ^)^. Our patients undergoing prosthetic surgery reported a greater intensity compared with that reported by trauma surgery patients. However, these results are not comparable to those of other studies, which compare different types of surgeries but not trauma with prosthetic surgery. Regarding pain treatment, although most postoperative patients receive some form of analgesic treatment, they continue to feel pain. In our study, the presence of postoperative pain during the PCU stay was a factor associated with the increased consumption of analgesics. The prescription of analgesics was similar to other studies, with NSAIDs and/or acetaminophen (alone or in combination) as the most used^(^ 11 ^,^ 17 ^)^. Morphine was used as rescue analgesia in almost a quarter of patients, similar to other studies ^(^ 3 ^)^. These data support the conclusion that pain remains inadequately treated with the current guidelines and that there is significant room for improvement.

Increasingly, emotional factors play an important role in postoperative pain. Hence, different authors have examined these factors in isolation, as predictors of postoperative pain during interventions of orthopedic and trauma surgeries^(^ 8 ^-^ 9 ^,^ 19 ^,^ 24 ^-^ 25 ^)^. Some authors have analyzed anxiety/depression or preoperative anxiety as predictors of postoperative pain^(^ 19 ^,^ 24 ^)^. Others have reported on the association between anxiety and pain^(^ 8 ^-^ 9 ^,^ 25 ^)^; although all of the investigators agree there is an association between these factors, it is unclear whether anxiety causes pain or pain causes anxiety. Our results point to other emotional factors besides anxiety. However, in the literature, there is no evidence of these aspects, which would enable a proper comparison. In our study, the results regarding anxiety are consistent with those of previous investigations and show that anxiety is a predictor of postoperative pain. However, these data are correlated with the immediate postoperative period. Additionally, the results of published studies are for the first 24 hours or 48 hours of the postoperative period or are the follow-up data of patients monthly or at one year after surgery. Furthermore, there is a significant degree of variability in the type of tools used to assess anxiety and pain, once more making it difficult to compare the data.

In reference to the study limitations, although this is a retrospective study, the outcomes are an updated approximation of the predictors of a high prevalence of postoperative pain. These data may serve as a starting point to encourage further research. A possible bias may exist due to the data and patient selection. However, the data obtained from the medical records was complete, without losses due to a lack of information in the main study variables. The use of our scale may limit the comparison of our results with those of other researchers because they used psychological assessment tools, while we preferred to use an instrument of routine clinical practice in our study. This instrument is a widely used tool to assess emotional factors, but it is not an instrument for a complete psychological evaluation, and its use cannot be interpreted in this regard. However, the available evidence on predictors of postoperative pain does not assess emotional factors such as fear, stress, tearing or sweating. Therefore, our data further elucidate the results published so far and show that the use of simple routine methods during the preoperative period may help identify patients with an increased risk of postoperative pain, with the possibility of individualized interventions when deemed necessary. Finally, the retrospective nature of the study may limit the interpretation of the results, but in our opinion, the present findings are a good first approximation to the issue. 

## Conclusion

In conclusion, the present investigation identified two predictors of immediate pain during the postoperative period: age and preoperative anxiety. Consequently, we recommend that young and anxious patients have customized analgesic regimens to better alleviate their postoperative pain within the first hours after surgery. The establishment of protocols with specific instruments for assessing and treating anxiety as a predictor of postoperative pain is encouraged. This intervention would help health professionals understand and improve the perception of their patients' pain and optimize pain treatment.
